# Comparison of CT-Guided Microwave Ablation of Liver Malignancies with and Without Intra-Arterial Catheter Placement for Contrast Administration

**DOI:** 10.3390/curroncol32010028

**Published:** 2025-01-02

**Authors:** Anne Bettina Beeskow, Holger Gößmann, Hans-Jonas Meyer, Daniel Seehofer, Thomas Berg, Florian van Bömmel, Aaron Schindler, Manuel Florian Struck, Timm Denecke, Sebastian Ebel

**Affiliations:** 1 Department of Diagnostic and Interventional Radiology, University Hospital Leipzig, 04103 Leipzig, Germany; anne.beeskow@medizin.uni-leipzig.de (A.B.B.); holger.goessmann@medizin.uni-leipzig.de (H.G.); hans-jonas.meyer@medizin.uni-leipzig.de (H.-J.M.); timm.denecke@medizin.uni-leipzig.de (T.D.); 2 Department of Hepatobiliary Surgery and Visceral Transplantation, University Hospital Leipzig, 04103 Leipzig, Germany; daniel.seehofer@medizin.uni-leipzig.de; 3 Department of Gastroenterology, Hepatology, Infectiology and Pneumology, University Hospital Leipzig, 04103 Leipzig, Germany; thomas.berg@medizin.uni-leipzig.de (T.B.); florian.vanboemmel@medizin.uni-leipzig.de (F.v.B.); aaron.schindler@medizin.uni-leipzig.de (A.S.); 4 Department of Anesthesiology and Intensive Care Medicine, University Hospital Leipzig, 04103 Leipzig, Germany; manuelflorian.struck@medizin.uni-leipzig.de

**Keywords:** microwave ablation of the liver, intra-arterial catheter placement, hepatic malignancies

## Abstract

Background: The aim of this study was to compare microwave ablation (MWA) with and without prior placement of an intra-arterial catheter for the purpose of application of contrast medium (CM). Methods: 148 patients (45 female, 65.1 ± 14.9 years) with liver tumors who underwent CT-guided MWA were included. Of these, 25 patients had an IA catheter placed in the hepatic artery. Results: 37 patients underwent planning imaging for MWA without CM. A total of 86 patients received a standard dose of 80 mL intravenous (IV) CM for the planning scans. The patients with an IA catheter (*n* = 25) received an IA application of 10 mL CM. A total of 29 patients received contrast-enhanced scans in the PV phase for control of needle positioning after IV application of a standard dose of 80 mL CM. In patients with an IA catheter, control of the needle position was performed by single-slice scans. IA CM application during the ablation enabled monitoring of the ablation zone. Over the entire intervention, patients with IA catheters received less CM as compared to patients without an IA catheter (39.1 ± 10.4 mL vs. 141 ± 39.69 mL; *p* < 0.001). Conclusions: IA catheter placement was associated with a significant decrease of the amount of CM during MWA and enabled monitoring of the ablation zone.

## 1. Introduction

Percutaneous, image-guided ablation techniques for primary and secondary liver malignancies such as microwave ablation (MWA) are now integral parts of modern multimodal oncological treatments [1,2]. The aim of MWA is to achieve sufficient tumor control with a safety margin of 0.5–1 cm in all directions with the best possible protection of healthy liver tissue [3]. To achieve this, reliable visualization of the target tumor is of utmost importance. If the tumor is not visible on native imaging, intravenous (IV) contrast agent applications and control scans can be performed repeatedly to visualize the tumor before, during, and after ablation. This technique may require the application of large amounts of contrast medium (CM). In the case of hepatocellular carcinoma (HCC), the tumor can be marked with lipiodol before ablation, which often is not possible in the case of secondary malignancies such as colorectal liver metastases [4]. Additionally, intra-arterial (IA) CM application has been shown to be feasible in terms of tumor visualization [5].

Complications in the context of MWA can be divided into heat-related and puncture-related complications [6]. Heat-related complications include, for example, secondary bile duct strictures, while puncture-related complications mainly include bleeding from the tumor or from accidentally injured arteries at the puncture site. Puncture-related bleeding can usually be treated endovascularly by embolization of the affected vessel or vascular territory, but this requires the placement of a diagnostic and microcatheter to the site first [7].

Due to the abovementioned aspects, there is a need for proper visualization of the target lesion. The purpose of the present study was to retrospectively analyze all MWA performed at a single center in order to elucidate whether IA catheter placement for the purpose of CM administration for tumor visualization can contribute to a reduction in the amount of CM. Furthermore, possible additional advantages such as better visualization of the tumor and the ablation zone during MWA and improved management of puncture-related bleeding complications, by having a diagnostic catheter already in place to perform an embolization procedure, were explored.

## 2. Materials and Methods

### 2.1. Study Design

In this retrospective study at a single center, we retrospectively examined all MWA procedures performed between January 2020 and August 2023 for previously untreated liver tumors. All procedures were performed by senior radiologists (>8 years of experience). Institutional ethics committee approval was obtained. Written informed consent was obtained from all patients. All methods were carried out in accordance with the relevant guidelines and regulations. All procedures were analyzed regarding the numbers of periprocedural CT scans, the amount of CM used and the occurrence of complications by screening the procedure images and reports, which was saved to our radiology information system (Medavis RIS, Karlsruhe, Germany). Additionally, the pre- and postinterventional serum creatinine levels were investigated.

### 2.2. Study Cohort

This analysis included 148 patients (45 female, mean age 65.1 ± 14.9 years) with primary liver tumors and metastasis who underwent CT-guided MWA between January 2020 and August 2023. Of these 148 patients, 25 were scheduled for CT-guided MWA between January 2023 and August 2023 with prior IA catheter placement for the purpose of IA application of CM during MWA. The decision for an ablation treatment had been made by an interdisciplinary tumor board.

### 2.3. Arterial Catheterization

All angiographies were performed on a flat panel system (Azurion Clarity IQ, Philips Healthcare, Best, The Netherlands). Catheter placement into the common hepatic artery was performed under local anesthesia via the right groin. Since the patients were scheduled for percutaneous ablation afterwards, no heparin was used for the catheterization. The right common femoral artery (CFA) was accessed using a 4F angiography sheath. A 4F catheter was placed in the common hepatic artery. In one case (4% of all cases), the catheter was placed into the right hepatic artery, which arose from the superior mesenteric artery. To prevent dislocation, the sheath and the catheters were attached to the skin using sterile adhesive strips. To ensure sterility, the puncture site, sheath, and catheter were covered with an additional sterile cloth. Then, the patients were transferred to the CT unit. After completion of the ablation, the catheters and sheaths were removed and a pressure bandage was applied over the puncture site, which remained in place for four hours.

### 2.4. Percutaneous MWA

The ablations were performed under general anesthesia or sedation and local anesthesia. All ablations were performed on a multislice CT scanner (Big Bore 16 Slice, Philips, Best, The Netherlands). For access route identification, a radiopaque marker was placed on the skin in the liver region. For all procedures, iodine-containing (300 mg/cc), non-ionic CM was used. The planning scan was either performed after IV application of a standard dose of 80 milliliters (mL) CM plus 30 mL NaCl or after intra-arterial (IA) application of 10 mL CM (300 mg/cc iodine) plus 30 mL NaCl with a scan delay of 5 s. IV CM application was performed with an infusion rate of 2–3 mL/second, and IA application was performed with a rate of 4 mL/second. Then, the microwave probe (AMICA, H.S. Hospital Service S.p.A. in Latina, Italia and NeuWave, Johnson and Johnson, New Brunswick, NJ, USA) was navigated without dedicated targeting systems into the tumor with single-slice image acquisitions after every needle manipulation. In the cases with an IA catheter, small amounts of CM (4 mL CM plus 16 mL NaCl) were applied during needle placement for visualization of the tumor and the intrahepatic arteries. Correct needle placement was either verified by single-slice image acquisitions (in case of IA catheters in place) or by acquisition of a helical scan of the target region with or without application of CM (in cases without IA catheters in place). After correct needle positioning, the ablation of the tumor with a safety margin of at least 5 mm in all directions was performed. Post-ablation imaging consisted of a scan either after IV application of 80 mL CM or after IA application of 10 mL CM plus 30 mL NaCl.

### 2.5. Statistical Analysis

All analyses were performed using SPSS statistical software V28 (IBM, Armonk, NY, USA). The distribution of the variables was tested using the Shapiro–Wilk test for normality. Once normally was proven, quantitative variables were expressed as mean values and standard deviations (SD). Analysis included Mann–Whitney U Test for continuous variables and paired *t*-test for categorical variables. A *p*-value < 0.05 was considered statistically significant.

## 3. Results

### 3.1. Study Cohort

All 148 datasets could be analyzed. Patients had either one (*n* = 83, 60.3%), two (*n* = 61, 37.6%), or three tumors (*n* = 4, 2.1%). MWA was performed under general anesthesia (*n* = 91, 67.2%) or sedation and local anesthesia (*n* = 57, 32.8%). The cohort consisted of 62 patients with hepatocellular carcinoma (HCC), 67 patients with colorectal rectal liver metastasis (CRLM), 11 patients with liver metastasis from cholangiocellular carcinoma (CCC), 4 patents with metastasis from melanoma, 2 patients with metastasis from neuroendocrine tumors (NET), and 2 patients with liver metastasis from lung cancer (LC). A total of 62 patients suffered from liver cirrhosis child A (*n* = 41), child B (*n* = 11), and child c (*n* = 10). All 148 tumors were untreated to the point of MWA. A total of 25 consecutive patients without any prior local treatment of the target tumor underwent MWA with prior intra-arterial catheter placement; among these patients were 18 with one (*n* = 16) or two (*n* = 2) HCC nodules, 6 patients with one (*n* = 5) or two (*n* = 1) CRLM, and one patient with one liver metastasis from melanoma. A total of 123 patients underwent MWA without an IA catheter. See Table 1.

### 3.2. Pre-Procedural Tumor Imaging Characteristics

All patients underwent either multiphasic CT or magnetic resonance imaging (MRI) for staging prior to tumor board discussion. All HCCs were hyperenhancing in the arterial phase with typical wash-out in the portal-venous (PV) phase. Metastasis from melanoma was hyperenhancing in the atrial phase and hypoenhancing in the PV phase. All CRLMs, CCCs, metastases from LC, and NET were hypoenhancing in the arterial phase and showed a rim-enhancement in the PV phase. Mean tumor diameter was 26 ± 14 mm (12–31 mm) without significant differences between the groups (with vs. without IA catheter) (*p* > 0.05).

### 3.3. Amounts of Contrast Medium

Throughout the intervention, patients with IA catheters received significantly less CM than patients without IA catheters (39.1 ± 10.4 mL vs. 141 ± 39.69 mL; *p* < 0.001). See Table 2.

### 3.4. MWA Planning Scans

Thirty-seven patients underwent planning imaging for MWA without administration of CM. A total of 86 patients received IV CM for the planning scans, of which in 38 patients biphasic (arterial and PV phase) and in 48 patients monophasic (PV phase) scans were obtained. The patients with an IA catheter (*n* = 25) received IA application of 10 mL CM plus 30 mL NaCl, and an arterial scan was obtained (Table 1). In patients with an IA catheter, all HCC was hyperenhancing and all CLRM and metastasis from melanoma showed a rim-enhancement.

### 3.5. Periinterventional Control Scans for Needle Positioning

**Figure 1:**
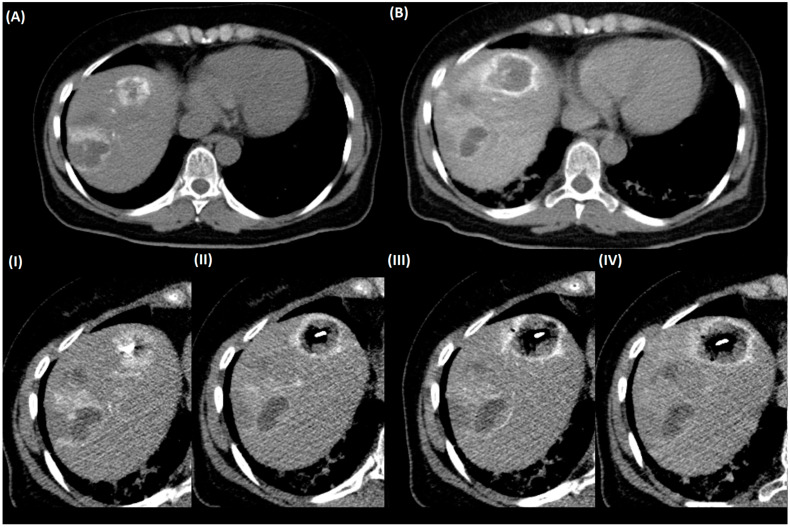
CT scan in a patient with contrast medium administration via an IA catheter in the common hepatic artery during ablation of a colorectal liver metastasis. (**A**) Planning scan before ablation, (**B**) final scan after ablation, (**I**–**IV**) control images during ablation with administration of 2–4 mL of contrast medium IA show growing of the ablation area.

Twenty-nine patients without an IA catheter received contrast-enhanced scans in the pv phase for control of needle positioning after IV application of a standard dose of 80 mL CM. In patients with an IA catheter control of the needle position was performed by single-slice scans. Mean CM dosage in patients with an IA catheter was 19.3 ± 4.1 mL. Intra-arterial CM application during the ablation enabled visualization of the ablation zone during heating of the needle (Figure 1). In 94 patients, no dedicated scans for control of the needle position were performed. (See Table 3).

### 3.6. Post Ablation Control Scans

In all patients, contrast-enhanced scans were performed for final control after the ablation. One-hundred-sixty-four patients therefore received a standard dose of 80 mL CM, and patients with an IA catheter (*n* = 25) received a standard dose of 10 mL CM plus 30 mL NaCl. (See Table 4).

### 3.7. Outcome and Complication Rates

Post-ablation imaging showed a safety margin of >5 mm in all treated cases, which translates to a technical success rate of 100%. In two cases (1.4%) (both without an IA catheter), post-ablation imaging showed a subcapsular hematoma with active contrast extravasation. Both patients were transferred to the angiography suite, and the bleedings were treated by coil-embolization of the injured hepatic artery. Three patients suffered from breathing-dependent pain in the right upper abdominal quadrant after ablation, which resolved after administration of analgesic medication within 12 h. Furthermore, there were no adverse events. Additionally, during a follow-up period of 24.1 ± 22.1 months, there was local tumor recurrence in two patients (1.4%), both without IA catheters.

### 3.8. Kidney Function

The pre- and post-interventional mean creatinine value [µmol/L] was 87 ± 24 and 92 ± 31 in the group without IA catheter and 84 ± 19 and 96 ± 25 in the group with IA catheter, in each case without significant differences (*p* = 0.63). Mean serum creatinine after 3 months was 83 ± 29 and 80 ± 34, respectively (*p* = 0.55). See Table 1.

## 4. Discussion

In this study, intra-arterial catheterization for the purpose of CM application was associated with a significant reduction of the amount of CM needed for ablation planning, needle placement, and post-ablation control. Furthermore, this technique enabled visualization of the ablation zone.

There are several recommendations from various institutions regarding the necessary quantity and concentration of iodine-containing CM for various CT protocols. For example, the thorax working group of the German Radiological Society recommends a quantity of 80–120 mL of iodine-containing contrast medium for staging examinations (thorax and abdomen) in the context of lung tumors for optimal parenchymal imaging [8]. The European Society for Gastrointestinal and Abdominal Radiology recommends an injection volume of 70–140 mL IV for adult patients with an iodine concentration of 200–400 mg/mL [9]. Additionally, the Cardiovascular and Interventional Radiological Society of Europe (CIRSE) standards of practice on thermal ablation of liver tumors highlights that immediate post-ablation scans are essential to demonstrate sufficient ablative margins that are strongly related to local tumor control [3,10,11]. At our institution, we use a standard dose of 80 mL of IV contrast for venous imaging in adult patients with optimal parenchymal contrast in the setting of thermal ablation, which may result in up to 240 mL of CM in patients receiving IV CM for the planning scan, the scan to confirm proper needle placement, and the post-ablation scan. In the present study, we were able to show that the insertion of an IA catheter allows a MWA to be performed with a planning scan, a control scan for needle position, and a final scan with a CM dose of 39.1 ± 10.4 mL, resulting in a CM dose reduction of up to 72% compared to an IV application. This can be particularly beneficial for patients who are at risk of developing kidney failure [12]. In our cohort, 29 patients in the pv phase received contrast-enhanced images (80 mL intravenously) to check needle positioning, while in patients with an IA catheter, these checks were performed using single-slice images after injection of small amounts of CM through the catheter, resulting in less radiation exposure for the patients. In addition, since oncology patients receive a high number of CT scans during their patient career, this can be particularly beneficial. On the other hand, it must be borne in mind that not all patients agree to have an IA catheter inserted, so patients must also be offered the procedure without an IA catheter.

MWA allows minimally invasive tumor therapy, sparing as much healthy liver tissue as possible while still achieving a sufficiently large ablation area to minimize the risk of local recurrence [1,2]. The size of the ablation area depends on the energy delivered by the probe, ablation time, and tissue-dependent factors such as thermal conductivity, proximity to large blood vessels, etc., which can make it difficult to predict the exact size of the ablation area [13]. The information provided by the manufacturer of the ablation system on ablation sizes usually comes from an animal model (mostly cadaver studies), so that this information can only serve as a guideline, which is why multiple new techniques are being tested to be able to accurately predict the size of the ablation area of the MWA [14,15,16,17]. While the size of the ablation area or the ice ball in cryoablation can be visualized directly in MRI, this possibility is lacking in MWA in CT [18]. In our study, we were able to observe the size of the ablation area during heating of the needle in patients with an IA catheter by injecting 2–4 mL of CM and acquiring single slices (see Figure 1). These results suggest that this technique could increase the precision of MWA. However, this has yet to be proven in randomized controlled trials.

Major complications during MWA such as puncture bleeding, e.g., due to injury to a hepatic artery can occur in 2.2–3.1% and can be treated by an endovascular approach [19,20]. In patients who have received an IA catheter in the common hepatic artery prior to MWA, this opens the window of opportunity for endovascular bleeding control in the event of a bleeding complication. This could save valuable time in an emergency situation and potentially minimize the adverse effects of bleeding.

Arterial angiography can lead to complications such as infection, bleeding, or vascular occlusion at the puncture site. For femoral punctures, the frequency of infections is stated as <1%, while the frequency of secondary bleeding is stated as 0.4–0.7% [21]. In the case of radial punctures, the risk of occlusion of the radial artery with possible ischemia of the hand must be taken into account, which according to the literature occurs in fewer than 0.3% of cases with correct preparation [22,23,24]. In our study, we used a femoral approach, which was closed after completion of the procedure by manual compression and application of a pressure bandage without complications. Due to the low complication rate reported in the literature, it would also be conceivable to use an approach from the radial artery with subsequent application of a compression bandage to increase patient comfort so that patients can be mobilized earlier after the procedure. Regarding the discussion about performing interventional radiology procedures on an outpatient basis, a radial artery approach might particularly be advantageous [25].

Since contrast media is a cost item, it can be advantageous to save on it in times of increasing financial pressure on hospitals. By placing an IA catheter, we were able to reduce the amount of contrast medium used by an average of approx. 100 mL per patient, which at a price of approx. EUR 1700 per L of contrast medium (estimated price at a university center) could mean a cost saving of around EUR 170, particularly in high volume centers or for patients requiring repeat procedures. The costs for placing the IA catheter must of course be calculated against this.

Limitations of the present study are its retrospective and single-center design, leading to a biased comparability of the groups (IA catheter vs. no IA catheter). Although no special selection of patients who received an IA catheter was carried out, all patients who were treated with MWA between January 2023 and August 2023 received an IA catheter, the comparability is nevertheless reduced compared to a randomized study design. The comparability is also limited due to the small cohort size of the IA catheter group. However, this study should rather be seen as a proof-of-concept and the results must be verified in future randomized studies. The retrospective design also means that there was no fixed study protocol. Although all procedures were performed by senior radiologists, it could be that the techniques differed to some extent. However, it can be argued that this is “real-world data”, which tends to enhance the strength of the results. Due to the retrospective design of this study, only limited conclusions can be drawn about the long-term results of the procedure. In the analyzed cohort, the recurrence-free survival is 24.1 ± 22.1 months (ongoing) without significant differences between the groups and without differentiation between tumor entities. Long-term data, besides 3-month serum creatinine, regarding the influence of the use of an IA catheter on renal function cannot be determined either. A systematic analysis of all aspects must be carried out in larger, prospective studies, for which a prospective study has already been designed and is ongoing, which also provides data regarding the combination of arterial contrasting with percutaneous ablation [26].

## 5. Conclusions

In conclusion, the present study suggests that the placement of an IA diagnostic catheter before MWA may be associated with a decrease of the amount of CM and therefore lower costs and allows for monitoring of the ablation zone without increasing the complication rate.

## Figures and Tables

**Table 1:** Patient baseline characteristics.

Cohort [*n*]	148	
Male [*n*/%]	103/71%	
Female [*n*/%]	45/29%	
Mean age [years]	65.1 ± 14.9	
Groups	Ablation with IA Catheter	Ablation without IA Catheter
[*n*]	25	123
Tumor entity [*n*]		
HCC	18	44
CRLM	6	61
CCC	0	11
Melanoma	1	3
NET	0	2
Lung cancer	0	2
Liver cirrhosis [*n*/%]	18	44
Stadium Child–Pugh Stadium A [*n*]	7	34
Stadium Child–Pugh Stadium B [*n*]	5	6
Stadium Child–Pugh Stadium C [*n*]	6	4
Serum creatinine [µmol/L]		
Pre-interventional	87 ± 24	84 ± 19
Post-interventional	92 ± 31	96 ± 25
After 3 months	83 ± 29	80 ± 34

**Table 2:** Overview of the distribution of the amount of contrast medium in patients who received either no contrast medium or intravenous or intra-arterial contrast medium for the planning scan before MWA.

	Non-Contrast Scan	Intravenous CM		Intra-Arterial CM
		Monophasic Scans	Bisphasic Scans	
Number of Patients *n*	37	48	38	25
Amounts of CM [mL]	0	80 + 30 mL NaCl		10 + 30 mL NaCl

**Table 3:** Overview of the distribution of the amount of contrast medium in patients who received either no contrast medium or intravenous or intra-arterial contrast medium to check the needle position during the MWA.

	No Dedicated Scan for Control of Needle Position	Intravenous CM	Intra-Arterial CM
		Monophasic Scans	
Number of patients [*n*]	94	29	25
Amounts of CM [mL]	0	80	19.3 ± 4.1

**Table 4:** Overview of the distribution of the amount of contrast medium in patients who received either intravenous or intra-arterial contrast medium for the final check after MWA.

	Intravenous CM	Intra-Arterial CM
	Monophasic Scans	
Numer of patients [*n*]	123	25
Amounts of CM [mL]	80 + 30 mL NaCl	10 + 30 mL NaCl

## Data Availability

The datasets used and/or analyzed during the current study are available from the corresponding author on reasonable request.

## Abbreviations

IA—intra-arterial; CM—contrast medium; MWA—microwave ablation; IV—intravenous; PV—portal venous; CT—computed tomography; HCC—hepatocellular carcinoma; mL—milliliters; SD—standard deviation; CRLM—colorectal rectal liver metastasis; CCC—cholangiocellular carcinoma; NET—neuroendocrine tumors; LC—lung cancer; cTACE—conventional transarterial chemoembolization; MRI—magnetic resonance imaging.
